# Optimization of Whey Protein-Based Films Incorporating *Foeniculum vulgare* Mill. Essential Oil

**DOI:** 10.3390/jfb14030121

**Published:** 2023-02-23

**Authors:** Salomé Pedro, Luísa Pereira, Fernanda Domingues, Ana Ramos, Ângelo Luís

**Affiliations:** 1 CICS-UBI, Health Sciences Research Centre, University of Beira Interior, Av. Infante D. Henrique, 6200-506 Covilhã, Portugal; 2 CMA-UBI, Centre of Mathematics and Applications, University of Beira Interior, Rua Marquês d’Ávila e Bolama, 6201-001 Covilhã, Portugal; 3 Chemistry Department, Sciences Faculty, University of Beira Interior, Rua Marquês d’Ávila e Bolama, 6201-001 Covilhã, Portugal; 4 FibEnTech-UBI, Fiber Materials and Environmental Technologies Research Unit, University of Beira Interior, Rua Marquês d’Ávila e Bolama, 6201-001 Covilhã, Portugal

**Keywords:** Box–Behnken, experimental design, whey protein, fennel, essential oil, packaging, antioxidant, antibacterial

## Abstract

Petroleum-based plastics used in food packaging are not biodegradable. They accumulate in the environment in large amounts, causing a decrease in soil fertility, jeopardizing marine habitats, and causing serious problems to human health. Whey protein has been studied for applications in food packaging, either because of its abundant availability or because it confers transparency, flexibility, and good barrier properties to packaging materials. Taking advantage of whey protein to produce new food packaging materials is a clear example of the so-called circular economy. The present work focuses on optimizing the formulation of whey protein concentrate-based films to enhance their general mechanical properties applying the Box–Behnken experimental design. *Foeniculum vulgare* Mill. (fennel) essential oil (EO) was incorporated into the optimized films, which were then further characterized. The incorporation of fennel EO in the films leads to a significant increase (*p* < 0.05) in peak elongation (from 14.03 to 31.61%) and tensile index (from 0.40 to 0.50 N.m/g). The optimized whey protein films were yellowish and very transparent (>90%). The results of the bioactive activities of the optimized films showed their ability to be applied as active materials for food packaging to improve the shelf-life of food products and also to prevent foodborne diseases associated with the growth of pathogenic microorganisms.

## 1. Introduction

Traditional plastics produced from fossil fuels are a group of several materials with broad properties, namely, resistance, flexibility, weightlessness, and low-cost production. These properties make them ideal for numerous consumer and industrial applications. Due to their versatility, plastics are important materials in the food packaging industry [1]. However, as most of these plastics are not biodegradable, they accumulate in the environment in large amounts, causing a decrease in soil fertility, jeopardizing marine habitats, and causing serious problems to human health [2]. Consequently, scientists and other stakeholders have been developing bio-based materials to replace traditional plastics, particularly in the food packaging sector [3].

Edible films consist of a thin layer of biopolymers (mainly proteins and polysaccharides) formed on the food surfaces or intended to wrap food products as primary packaging. Depending on their composition, these films may have additional benefits, such as antioxidant and antimicrobial activities, which promote the extension of the shelf-life of packaged food [4].

The application of whey protein in food packaging has been studied, either because of its abundant availability (50 million tons of untreated whey/year) or because it confers flexibility, transparency, and great barrier properties (oxygen, aroma, and lipids) to packaging materials [5]. Whey is considered a waste of cheese manufacture and is usually discarded; however, its high protein content (isolate or concentrate) makes it very attractive for the development of active biopolymers for application in packaging [6]. Moreover, whey proteins have several bioactivities for the food industry and human health, they are currently recuperated from whey by using diafiltration and ultrafiltration processes [7]. Whey protein isolate (WPI) comprises about 90% protein and is the typical commercial product of whey protein. The main components of WPI are α-lactalbumin and β-lactoglobulin [7]. Whey protein concentrate (WPC) can be provided at a considerably lower cost than WPI, with benefits from both industrial and economic points of view [6]. Taking advantage of whey protein to produce new food packaging materials is a clear example of the so-called circular economy, which involves the closing of material loops or cascading used resources, to prevent waste from occurring, and transforming the resulting residual streams into new (secondary) resources [8]. Furthermore, whey protein films have been shown to be good vehicles for antioxidant and antimicrobial agents [9].

To enhance the characteristics of films intended for food packaging, different bioactive agents can be incorporated to confer antioxidant and/or antimicrobial properties. *Foeniculum vulgare* Mill. (fennel) is a perennial plant belonging to the *Apiaceae* family. Fennel presents several applications, including in the cosmetics industry and medicine. Current works have shown that fennel essential oil (EO) has bioactive properties, namely antioxidant and antimicrobial [10,11,12], which could represent a potential application in the development of bioactive packaging.

To increase the flexibility and processability of films used in food packaging, plasticizers are used. Plasticizers are a crucial category of non-volatile low molecular weight compounds broadly employed in the polymer industry as additives. Examples of plasticizers commonly used for the production of edible films include sorbitol and glycerol [13].

The present work focuses primarily on optimizing the formulation of whey protein concentrate-based films to improve their overall mechanical properties using the Box–Behnken experimental design. Afterwards, fennel EO was incorporated into the optimized whey protein films, which were then further characterized.

## 2. Materials and Methods

### 2.1. Reagents

The WPC (unflavored) powder was purchased from My Protein, a THG Company (Voyager House, Manchester, UK). Glycerol (anhydrous extra pure) (CAS Number: 56-81-5) was supplied by Merck (Darmstadt, Germany). *D*-Sorbitol (CAS Number: 50-70-4) was obtained from Aldrich (St. Louis, MI, USA).

### 2.2. Plant Material

Fennel (*Foeniculum vulgare* Mill., *Apiaceae*) EO was produced by Herdade de Vale Côvo (Alentejo, Portugal). The EO was obtained by steam distillation in a stainless-steel alembic from the aerial parts (flowers and leaves) of the plant that grows naturally in the field (organic farming, PT-BIO-02, ECOCERT), which were harvested by hand immediately after the flowering in the Spring of 2021. A voucher specimen is always archived by the producers.

### 2.3. GC-MS Analysis

Using the established technique ISO 7609:1985 [14], gas chromatography (GC) coupled with mass spectrometry (MS) was used to analyze the chemical composition of fennel EO. A DB_WAX UI column (Agilent, Santa Clara, CA, USA) (60 m × 0.25 mm × 0.5 m) and an Agilent 5977B MS (Santa Clara, CA, USA) detector fitted with an Agilent 7820A GC-FID (Santa Clara, CA, USA) were utilized. The oven was set for 6 min at 50 °C, 2 min at 190 °C, 4 min at 220 °C, 10 min at 220 °C, 4 min at 240 °C, and lastly 10 min at 240 °C. Helium served as the carrier gas with injection volumes of 0.1 µL for both at head pressures of 33 Psi (FID) and 25.5 Psi (MSD).

### 2.4. Preparation of Whey Protein Films

Initially, 3 g of WPC were dissolved in 100 mL of distilled water at room temperature for 15 min with magnetic stirring. Then, a mixture of plasticizers (1.9688 g of glycerol and 0.6563 g of sorbitol) was included in the initial solution that was kept under magnetic stirring for another 15 min at room temperature. The pH of the filmogenic solution was ascertained to 7.5 followed by 30 min in a water bath at 90 °C under magnetic stirring. Next, the solution was cooled with ice for 10 min and finally, 13.5 mL were added to polystyrene Petri dishes. The solvent casting method was used to obtain the films after 18 h in an aerated oven at 60 °C.

The fennel EO was incorporated into the optimized films. For that, after cooling the filmogenic solution, 0.6 g of fennel EO (20 %, *v*/*v* relative to the WPC) was added and kept at room temperature under magnetic stirring for 10 min. Then, this mixture was homogenized using an Ultra-Turrax homogenizer (IKA T25 Digital, Staufen, Germany) at 10,000 rpm for 5 min. Finally, the mixture was degasified under vacuum.

### 2.5. Optimization of the Films’ Formulation: Experimental Design

The main effects, interaction effects, and quadratic effects of the process parameters (WPC, glycerol, and sorbitol) on the physical (grammage and thickness) and mechanical (peak elongation, tensile index, and elastic modulus) properties of the whey protein-based films were statistically studied and evaluated using the Box–Behnken statistical screening design. The Box–Behnken design was chosen specifically because, for three variables, it necessitates fewer runs than a central composite design. The “missing corners” enable the experimenter to stay away from the combined factor extremes. Its cubic design is characterized by a group of points sitting at the midpoint of each edge of a multidimensional cube and center point repeats. In those circumstances, this characteristic prevents a potential loss of data [15,16]. The suitable ranges of WPC, glycerol, and sorbitol were preliminary identified based on a single-factor experiment for the creation of whey protein-based films. Each independent variable had three levels of coding, ranging from −1 to +1. The details are listed in Table 1.

Five center points were used in a total of 17 tests using the Box–Behnken method, and the responses for each experimental condition are shown in Table 2.

To correlate the link between the independent variables and the responses, and to identify the pertinent model terms, a second-order polynomial model fitting the Box–Behnken design was fitted by using the statistical software Design-Expert Version 13 (https://www.statease.com/software/design-expert/, StatEase Inc., Minneapolis, MN, USA) [15,16]. The least squares method was used to examine the data from the experimental design. Using the same software, the *F*-test (*p* ≤ 0.05) was used to determine the significance of the regression coefficients. The coefficient of determination R^2^ was used to represent how well the polynomial model equation fit the data, and the values of adjusted-R^2^ were assessed to ensure that the models were adequate. The estimation of goodness of fit in each case was based on the importance of each term in the equation. The ANOVA test was used to evaluate the outcomes. The fitted polynomial equations were statistically calculated, and 3D surface plots and contour plots were produced from the results to illustrate the link between the response and the experimental values of each element [15,16].

### 2.6. Grammage, Thickness, Mechanical, and Optical Properties

According to ISO 536:1995 [17], the ratio of the films’ mass to area (g/m^2^) was used to determine their grammage. Using an Adamel Lhomargy Model MI 20 digital micrometer and five random measurements, the films’ thickness (µm) was determined in accordance with ISO 534:2011 [18]. Peak elongation (%), tensile index (N.m/g), and elastic modulus (MPa) of the films were measured using a tensile tester (Thwing-Albert Instrument Co., West Berlin, NJ, USA), with the crosshead speed changed to 10 mm/min and the initial grasp changed to 50 mm while still following ISO 1924/2:2008 [19] with some modifications. The color coordinates (L*, a*, and b*) and transparency of the films were measured using a Color Touch 2 spectrophotometer. The measurements were carried out using an observation angle of 10° and the illuminant D65 (daylight with a UV component), taking into account a number of random places on the films [20].

### 2.7. Barrier Properties

Following ASTM E96-00 [21], the water vapor permeability (WVP) (g/Pa.day.m) and the water vapor transmission rate (WVTR) (g/m^2^.day) were calculated. To do this, test cups containing 15 g of anhydrous CaCl_2_ as desiccant that had previously been dried at 105 °C were sealed with the films. The cups were then set at a temperature of 23 ± 2 °C and a relative humidity (RH) of 50 ± 5%, and the weight discrepancies were monitored every 2 h for a period of 48 h. The gradient was calculated using the linear regression’s slope of increasing weight with time [20]. The oxygen transmission rate (OTR) (cm^3^/m^2^.day) was evaluated using the equipment Labthink PERME^®^ OX2/23 (Labthink International, Medford, MA, USA) in accordance with ISO 15105-2:2003 [22]. The diffusion cell received the fixed films. After injecting pure oxygen into the diffusion cell’s exterior chamber, the rate at which it permeated the films was monitored until a steady state was reached. By normalizing OTR with the oxygen pressure (1 atm) and the film thickness, oxygen permeability (OP) (cm^3^.m/m^2^.day.kPa) was determined [16]. Moreover, the films’ oil permeability (OilP) was determined. To do so, test tubes were filled with 5 mL of edible vegetable oil and sealed with the films. On top of a weighted filter paper, the tubes were placed upside down. The weight differential of the filter paper, the film thickness, the effective contact area, and the storage time (24 h) were used to determine the OilP (g.mm/m^2^.day) [23].

### 2.8. Contact Angles and Surface Free Energy

The sessile drop contact angle method was utilized to determine the contact angles of the films using a model OCAH 200 DataPhysics Instruments (Filderstadt, Germany) that allowed simultaneous image recording and data analysis. By determining the contact angles with three reference liquids (ethylene glycol, water, and di-iodomethane), the surface free energy (total, dispersive, and polar components) of the films was estimated. The surface tension elements of the reference liquids were provided by the equipment’s software. For each liquid and sample, contact angles were acquired from at least six observations. Surface free energies of the films were then calculated using the Owens, Wendt, Rabel, and Kaelble empirical model (OWRK) [20].

### 2.9. Thermal Analysis

Differential Scanning Calorimetry (DSC) thermograms of the films were obtained using a calorimeter Netzsch DSC 204 (GWP, Munich, Germany), operating at a heating rate of 5 °C/min and a temperature range of 25 to 350 °C. Samples of the films were kept at 105 °C for 24 h prior to the study to completely evaporate the free water and establish the corresponding baselines [20,23].

### 2.10. Antioxidant and Antibacterial Activities

The ability of the films to inhibit lipid peroxidation was assessed using the β-carotene bleaching test. The first step involved mixing 50 µL of a β-carotene solution (20 mg/mL of chloroform) with 40 µL of linoleic acid, 400 µL of Tween 40, and 1 mL of chloroform before the chloroform was further evaporated in a rotary evaporator. The leftover material was then mixed with 100 mL of distilled water that had been infused with oxygen to create an emulsion. Afterwards, 3 disks of the films (6 mm in diameter) were mixed with 5 mL of this emulsion. The mixtures were then placed in a water bath at 50 °C for one hour. At 470 nm, the samples’ absorbances were evaluated in comparison to a blank made with an emulsion without β-carotene [20].

The antibacterial activity of the films against seven foodborne pathogens (*Staphylococcus aureus* ATCC 25923, *Listeria monocytogenes* LMG 16779, *Enterococcus faecalis* ATCC 29212, *Bacillus cereus* ATCC 11778, *Salmonella* Typhimurium ATCC 13311, *Escherichia coli* ATCC 25922, and *Pseudomonas aeruginosa* ATCC 27853) was evaluated by solid assay The bacterial species were maintained with 20 % (*v*/*v*) glycerol at −80 °C, which were overnight grown in brain–heart infusion agar (BHI) 24 h before the antibacterial assays. Several bacterial colonies were suspended in a sterile saline solution (NaCl, 85%, *w*/*v*) for the solid assay, with the turbidity of this suspension being adjusted to 0.5 McFarland (1.5 × 10^8^ colony-forming units (CFU)/mL). Cut 6 mm-diameter disks of the films were positioned on inoculated Müeller–Hinton agar (MHA) or BHI plates. The plates were then incubated for 18 h at 37 °C. Following incubation, their diameters were measured using a digital pachymeter, and inhibitory zones were visually inspected [20].

### 2.11. Statistical Analysis

In general, the data were presented as median and range. The statistical application SPSS version 28 (https://www.ibm.com/spss, IBM, Portsmouth, UK) was used to analyze the data. For the continuous variables, differences between medians were accessed using the Mann–Whitney U test. The threshold for statistical significance was set at *p* < 0.05.

## 3. Results and Discussion

### 3.1. Chemical Composition of Fennel EO

The chemical composition of fennel EO studied by GC revealed that most of the compounds present are monoterpenes, with fenchone (27.58%), *trans*-anethole (24.81%), and limonene (21.32%) being the three major compounds (Table 3). Previous studies also reported that these compounds are present in fennel EO from Tunisia, France [24], and Tajikistan [12]. The chemical analysis of fennel EO allowed us to identify 97.67% of its chemical composition (Table 3).

The antibacterial and antioxidant properties of fennel EO were screened in preliminary work and the results demonstrated that it was effective at preventing lipid peroxidation and some Gram-positive bacteria that are known to cause foodborne illnesses from growing (data not shown).

### 3.2. Box–Behnken Analysis

The Box–Behnken experimental design was used to guide the tests, which examined the individual and combined impacts of the independent variables (WPC, glycerol, and sorbitol) on the physical (grammage and thickness) and mechanical (peak elongation, tensile index, and elastic modulus) properties of the films (Table 2).

To evaluate the sufficiency and fitness of the models, the values of the coefficient of determination (R^2^) and adjusted R^2^ were calculated (linear, interactive, quadratic, and cubic). The estimated R^2^ values for grammage, thickness, peak elongation, tensile index, and elastic modulus were 0.9662, 0.9861, 0.2599, 0.9980, and 0.9985, respectively (Table 4).

By accounting for the sample size and the number of terms in the model, the adjusted R^2^ value adjusts the R^2^ value. The adjusted R^2^ values for grammage, thickness, tensile index, and elastic modulus were 0.9297, 0.9683, 0.9955, and 0.9967, respectively (Table 4). According to the ANOVA results for grammage, thickness, tensile index, and elastic modulus, the generated quadratic models significantly affect the responses with *F*-values of 264.92, 55.28, 393.57, and 534.46, respectively. Nonetheless, a linear model was the best suitable for peak elongation (Table 4).

The experimental findings of the Box–Behnken experimental design were fitted with the second-order polynomial equation. To better understand the interactive correlation between the answers and process variables, five empirical models were built. Following are the final equations determined in terms of coded components and considering the outcomes shown in Table 5:

$$\begin{array}{cc} Grammage= & 129.1+11.369X_{1}+9.665X_{2}+5.394X_{3}-2.450X_{12}-0.823X_{13}\\ & -2.195X_{23}+0.946X_{1}^{2}-5.721X_{2}^{2}-6.339X_{3}^{2} \end{array}$$

$$\begin{array}{cc} Thickness= & 94.15+14.56X_{1}+3.91X_{2}+2.083X_{3}+4.868X_{12}-3.533X_{13}\\ & +2.189X_{23}+1.789X_{1}^{2}+1.479X_{2}^{2}+2.809X_{3}^{2} \end{array}$$

$$Peakelongation=27.483-1.660X_{1}+1.633X_{2}+2.815X_{3}$$

$$\begin{array}{cc} Tensileindex= & 0.550+0.165X_{1}-0.318X_{2}-0.085X_{3}-0.100X_{12}+0.005X_{13}\\ & -0.025X_{23}+0.038X_{1}^{2}+0.278X_{2}^{2}+0.123X_{3}^{2} \end{array}$$

$$\begin{array}{cc} Elasticmodulus= & 9.530+4.489X_{1}-8.399X_{2}-2.418X_{3}-3.053X_{12}\\ & -0.820X_{13}-1.445X_{23}+0.971X_{1}^{2}+7.946X_{2}^{2}+2.799X_{3}^{2} \end{array}$$

X_1_ is the concentration of WPC, X_2_ is the concentration of glycerol, and X_3_ is the concentration of sorbitol. X_12_, X_13_, and X_23_ are interactions between the independent variables.

To ensure that the residuals follow a normal distribution, data were evaluated. The residual gives the difference between the observed value of a response measurement and the value that is fitted under the theorized model, and the small residual value indicates that model prediction is accurate [16]. The normal probability plot represents the normal distribution of the residuals.

**Figure 1:**
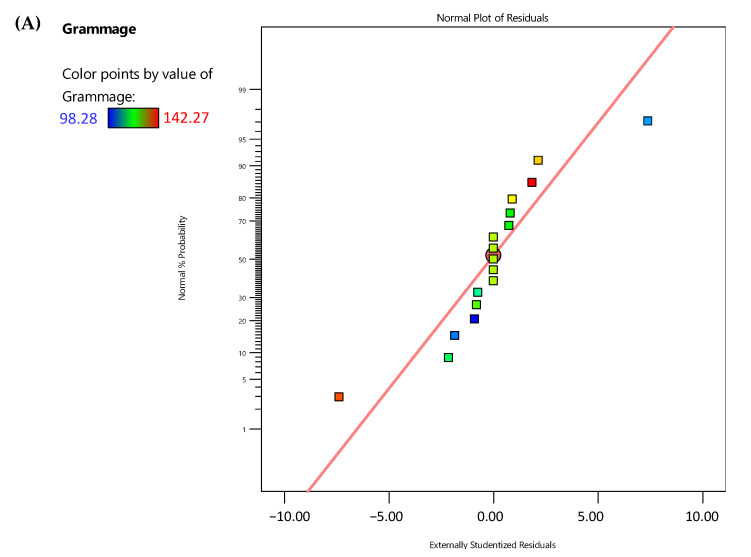

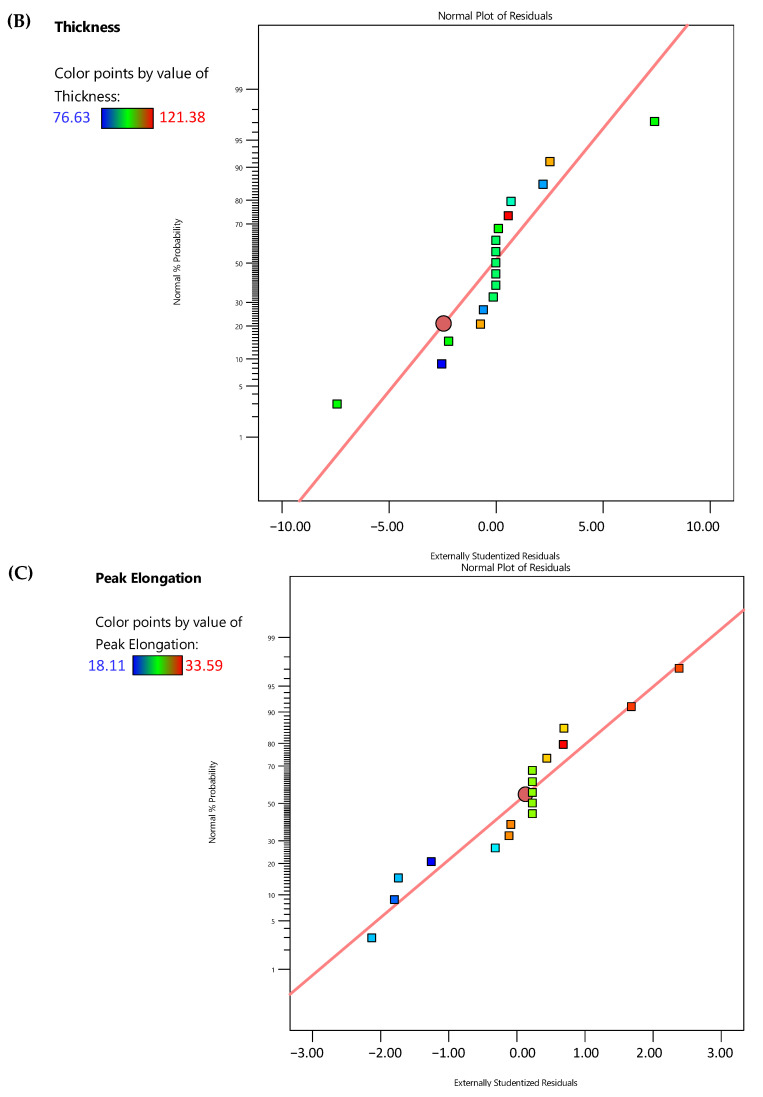

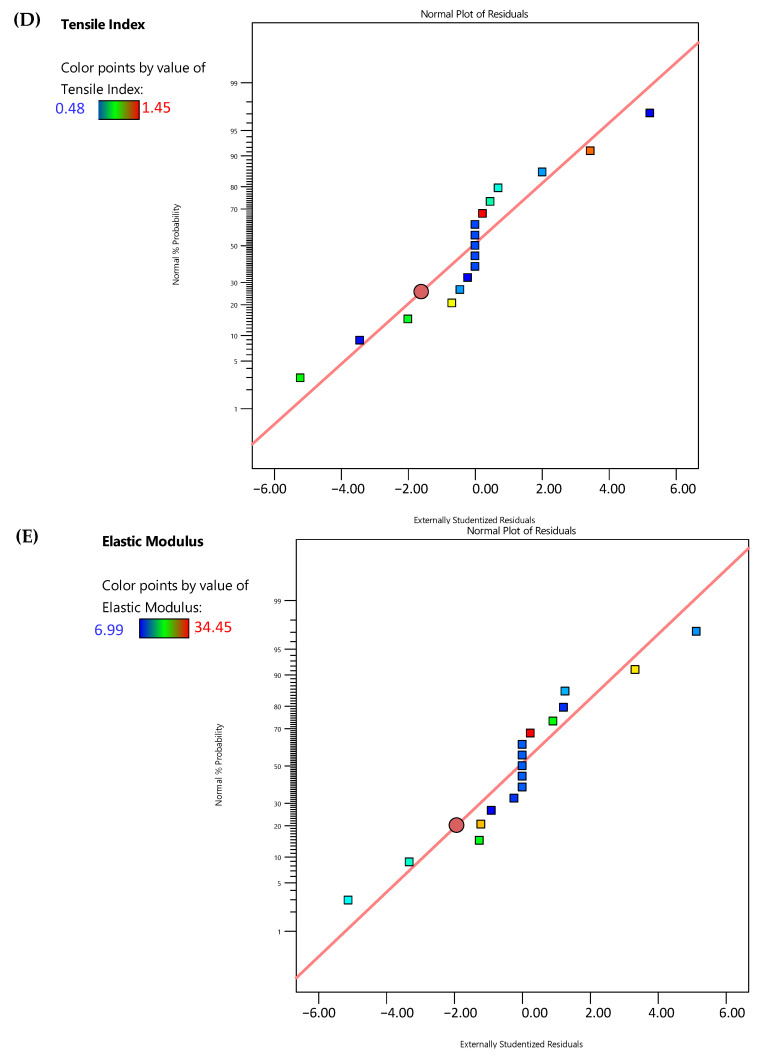
Normal probability plots of studentized residuals for grammage (g/m^2^) (**A**), thickness (µm) (**B**), peak elongation (%) (**C**), tensile index (N.m/g) (**D**), and elastic modulus (MPa) (**E**).

Figure 1 shows the graphs of the normal probability plots of the residuals, with a verification of the assumption of normality. In these graphs, it can be observed that the data points on the plots are reasonably close to a straight line, although some dispersion was expected, as previously mentioned by other authors [16].

**Figure 2:**
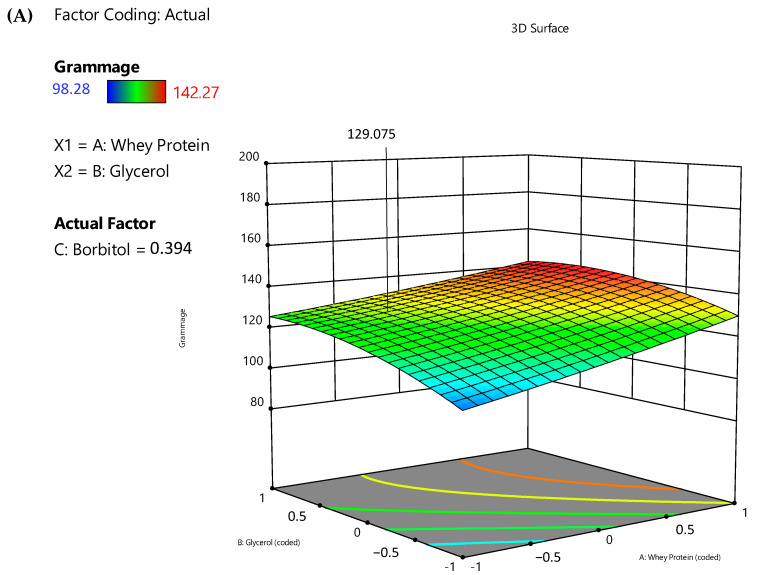

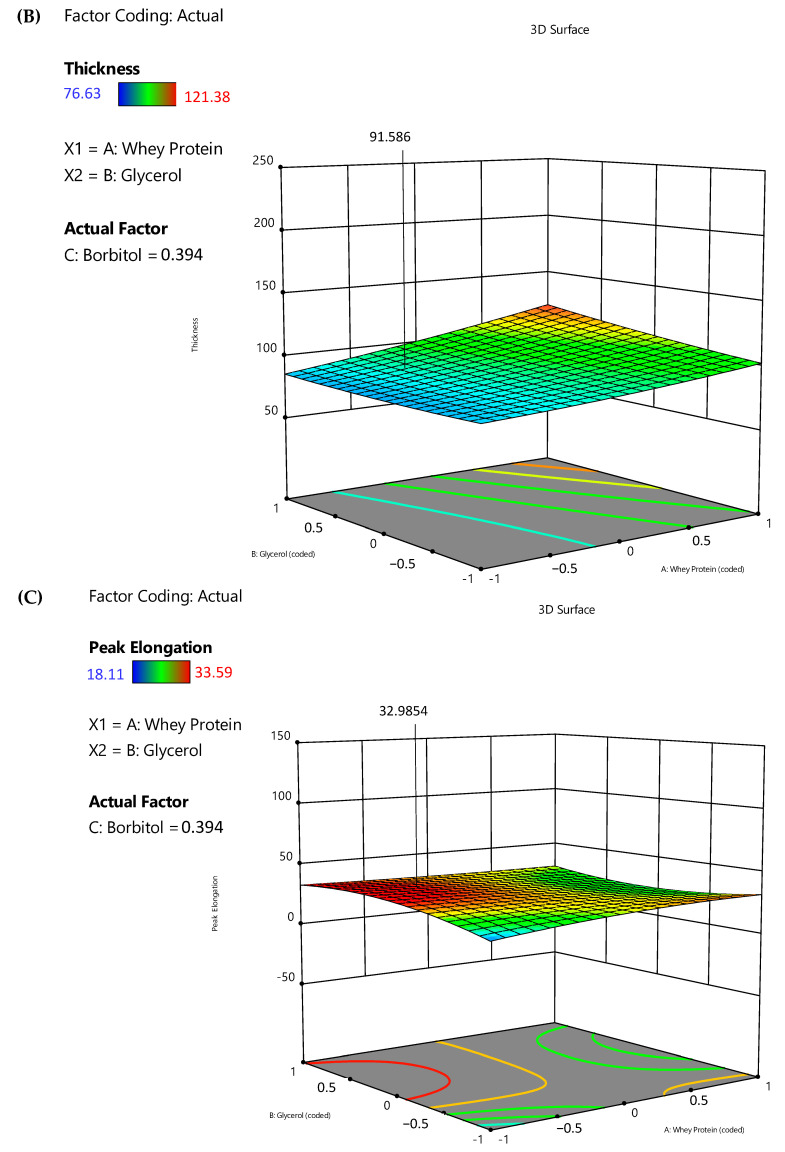

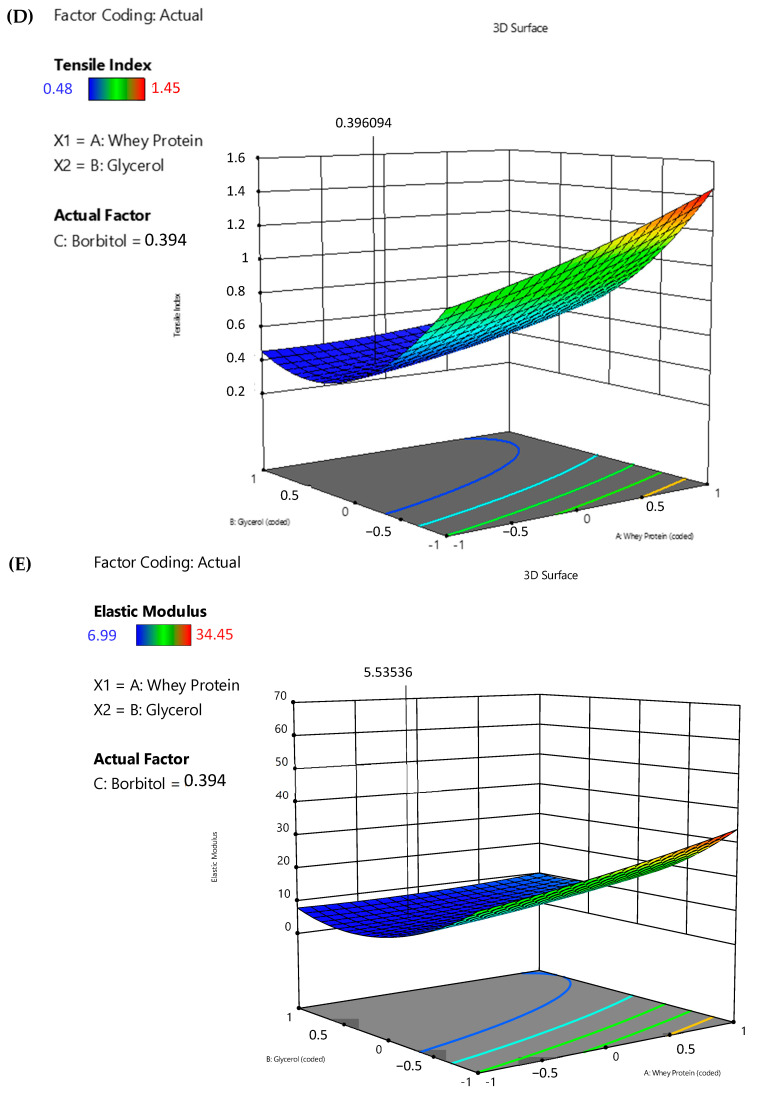
Response surface plots showing the interaction effects of process variables on grammage (g/m^2^) (**A**), thickness (µm) (**B**), peak elongation (%) (**C**), tensile index (N.m/g) (**D**), and elastic modulus (MPa) (**E**).

By observing the response surface plots showing the interaction effects of process variables (Figure 2), it was possible to verify that grammage ranged from 98.28 to 142.27 g/m^2^ due to variations in the concentration of WPC and glycerol (Figure 2A). Similar results were observed for the thickness of the films, which ranged from 76.63 to 121.38 µm (Figure 2B). The results obtained in this work are in line with what is expected because as the solid content of the filmogenic solutions increases, both the grammage and the thickness of the films increase proportionally.

The concentration of plasticizers (glycerol and sorbitol) directly affects the mechanical properties of the films. The results for peak elongation (18.11 to 33.59%) (Figure 2C), tensile index (0.48 to 1.45 N.m/g) (Figure 2D), and elastic modulus (6.99 to 34.45 MPa) (Figure 2E) were impacted depending on the concentration and proportion of glycerol and sorbitol.

Protein-based edible films have a high capacity for establishing many links and can make bonds in a variety of places. The capacity of a plasticizer to plasticize a protein-based polymer depends on several factors, including the molecular weight, quantity, and locations of its hydroxyl groups [13]. The mechanical resistance of films made with whey protein may decrease and film solubility in water may increase as glycerol content increases. Conversely, due to structural resemblances, the plasticizing impact of sorbitol in protein-based films may not result in appreciable variations in film properties when injected at the same molecular concentration of glycerol [13].

The optimal level of the components of the films was obtained after using the technique of the desired function, indicating that 2.774 g of whey protein, 2.1 g of glycerol, and 0.7154 g of sorbitol provide 129.075 g/m^2^ of grammage, 91.588 µm of thickness, 30.205 % of peak elongation, 0.3961 N.m/g of tensile index, and 5.535 MPa of elastic modulus. This set of optimum conditions was used to experimentally validate the model. To compare the experimental results with the expected values of the reactions using the derived empirical model equations, triplicate experiments were carried out. The experimental values obtained were 129.27 g/m^2^ for grammage, 94.00 µm for thickness, 14.03% for peak elongation, 0.40 N.m/g for tensile index, and 14.86 MPa for elastic modulus. The obtained results for grammage, thickness, and tensile index were found to be in agreement with the predicted values and clearly showed the suitability of the developed quadratic models. However, the obtained results for peak elongation and elastic modulus were not in agreement with the predicted values. Although the software suggested a linear model for peak elongation, it presented an R^2^ value of 0.2599 and a *p*-value of 0.2257, which indicates the weakness of this model to predict the peak elongation of the whey protein-based films. This may explain the discrepancy between the predicted and obtained values for peak elongation. Since the elastic modulus is experimentally calculated by the ratio between the stress and strain of the films and considering that the elongation is not well predicted by the developed model, it was expected that the predicted value for elastic modulus will also be affected in the prediction, which was verified.

### 3.3. Grammage, Thickness, Mechanical, and Optical Properties

After the development of the model for optimizing the whey protein-based films, these were produced to be further characterized. Moreover, fennel EO was incorporated into these films aiming at their functionalization, giving them antioxidant and antibacterial properties.

The results of the grammage and thickness of the films are presented in Table 6. The incorporation of fennel EO did not affect film grammage and thickness (*p* > 0.05). As reported by previous studies, the dry mass of each film solution (with or without fennel EO) in the Petri dishes during the casting step was almost the same [25]. Therefore, any differences in the mechanical properties of the films will not be due to differences in grammage or thickness. Most likely, the major effect of incorporating fennel EO will be due to chemical interactions that will be established between the whey protein molecules and the compounds of the EO.

As edible films are frequently exposed to several external forces during handling and food packaging, it is important to evaluate their mechanical properties [25]. Peak elongation, tensile index, and elastic modulus are commonly used parameters when studying the mechanical behavior of biopolymer films (Table 6). It was observed that the incorporation of fennel EO in the films leads to a significant increase (*p* < 0.05) in peak elongation (from 14.03 to 31.61%), and in the tensile index, thus demonstrating the plasticizer effect of fennel EO. The increase in these parameters may be related to the chemical structure of the compounds present in the EO. As these compounds are smaller than the whey protein chains, they can be intercalated between the whey protein molecules, thus increasing the flexibility and mechanical resistance of the films. Similar results for the mechanical properties of whey protein-based films were reported previously [26]. Other researchers who developed bioactive packaging made of whey proteins and EOs extracted from Thymbra (*Satureja capitata* L.) also noted a noticeable decrease in the film elastic modulus value and an appreciable increase in the peak elongation at the highest EO concentration tested, which obviously showed an increased plasticizing effect triggered by the EO [27].

The optical properties influence the general appearance and potential applications of the films, particularly their transparency [25]. The optimized whey protein films were yellowish and very transparent (>90%) (Table 6). Moreover, the incorporation of fennel EO did not influence significantly (*p* > 0.05) the optical properties of the films (Table 6).

### 3.4. Barrier Properties

The barrier properties of edible films are of major importance considering both the protection of packaged foods from environmental conditions and also the avoidance of migration of food components to the outside of packaging materials [25]. The barrier properties (water vapor, oxygen, and oil) of the whey protein-based films are summarized in Table 7. The incorporation of fennel EO has no impact (*p* > 0.05) on the barrier to water vapor (WVTR and WVP) (Table 7). These films are supposed to be hydrophilic and for that reason, it was expected that water vapor would be able to permeate the films [27]. Concerning the barrier to oxygen, the fennel EO leads to a significant increase (*p* < 0.05) in OTR and OP (Table 7). The entrapment of fennel EO compounds in the whey protein chains could promote a less compact polymeric network in the films that have hindered oxygen molecules to permeate through them. The barrier to oil (OilP) was not significantly affected (*p* > 0.05) by the incorporation of fennel EO (Table 7). Although the developed films, due to their hydrophilic character, do not seem to be a highly effective barrier to water vapor, they seem to represent a good barrier to hydrophobic substances.

### 3.5. Contact Angles and Surface Free Energy

The hydrophobic or hydrophilic properties of the films can be ascertained by measuring the water contact angle. Hydrophobic surfaces have water contact angles greater than 90°, and hydrophilic surfaces have values less than 90° [28].

Furthermore, measuring the contact angles with two more reference liquids (ethylene glycol and diiodomethane) allows us to determine the surface free energies of the films. Considering the results of contact angle measurements (Table 8), it was possible to conclude that the optimized whey protein-based films were hydrophilic, as expected, and confirming the results obtained for barrier properties, particularly to water vapor. Moreover, the fennel EO had no significant effect (*p* > 0.05) on water contact angle values (Table 8). Previous reports on water contact angles of whey protein-base films showed values ranging from that 46.69 to 56.61°, which are similar to the ones obtained in the present work [28]. The incorporation of fennel EO only decreased significantly (*p* < 0.05) the diiodomethane contact angle (Table 8).

### 3.6. Thermal Analysis

DSC analysis was utilized to measure the heat amount related with the thermal denaturation of the molecules in the films made from whey protein, which has also been used to evaluate the thermal stability of proteins [29].

**Figure 3:**
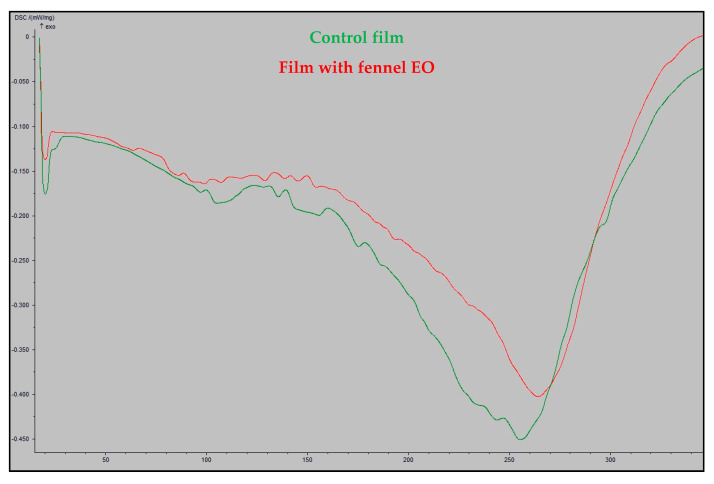
DCS thermograms of the whey protein-based films.

Both types of films displayed comparable thermal behavior in the DSC thermograms of the films (Figure 3). The endothermic peaks between 70 °C and 120 °C show that the whey proteins have begun to denature because of losing their layer of hydration. Moreover, the breakdown of whey proteins is shown by the endothermic peak above 220 °C. The deterioration of multicomponent materials, where more thermally stable bonds would need higher energies to dissolve, can be explained by these sets of peaks, which were similar to those reported in the literature [29,30].

### 3.7. Antioxidant and Antibacterial Activities

In food packaging, the antioxidant potential of the films is an important parameter for extending the shelf-life of the packaged food [31]. The antioxidant activity of the optimized whey protein-based films was evaluated by the β-carotene bleaching test, which allows us to estimate the capacity of the films to inhibit lipid peroxidation [32]. The results indicated that both types of films were able to inhibit lipid peroxidation (≈18%) and that the whey protein possesses intrinsic antioxidant activity, since the incorporation of fennel EO did not increase significantly (*p* > 0.05) the antioxidant activity of the films (Table 9). Similarly, other proteins, such as zein, have shown intrinsic antioxidant properties [33].

The inhibitory effects of the whey protein-based films on the growth of several foodborne pathogens were evaluated (Table 9). Films without EO showed contact inhibition against *E. coli* ATCC 25,922 and *P. aeruginosa* ATCC 27853, while films incorporated with fennel EO showed the ability to inhibit the growth of *S. aureus* ATCC 25923, *L. monocytogenes* LMG 16,779 and *E. faecalis* ATCC 29,212 (Table 9). A previous study also reported that whey protein films have the capacity to inhibit the growth of *Campylobacter jejuni* on inoculated turkey meat wrapped with the films during storage at 16 °C for 6 days [9]. Furthermore, other authors observed that whey protein films containing Thymbra EO showed a strong antimicrobial activity towards *Salmonella enteriditis* and *Salmonella enterica,* as well as against *S. aureus* ATCC 29213. These authors also observed a zone of inhibition surrounding the films activated with the EO, thus suggesting the ability of EOs to diffuse from the films into the agar matrix [27].

The results of the bioactive activities of the optimized films showed their potential to be used as active food packaging materials to improve the shelf-life of food products and also to prevent foodborne diseases associated with the growth of pathogenic microorganisms.

## 4. Conclusions

The Box–Behnken experimental design proved to be a useful tool in the formulation of the films, regarding the amount of whey protein concentrate and the concentration of plasticizers (glycerol and sorbitol). Moreover, the developed empirical models successfully predicted the values of grammage, thickness, and tensile index of the optimized whey protein-based films.

The incorporation of fennel essential oil improved the overall mechanical properties of the films as well as improved their antimicrobial properties by inhibiting the growth of several foodborne pathogens.

## Figures and Tables

**Table 1:** Experimental range and levels of the independent variables.

Initial Protocol	Variables (g)	Coded Variables	Variable Levels			Step Change Value ΔZ_1_
			−1	0	+1	
3	WPC	X_1_	2.5	3	3.5	0.5
1.9688	Glycerol	X_2_	1.7188	1.9688	2.2188	0.25
0.6563	Sorbitol	X_3_	0.5063	0.6563	0.8063	0.15

**Table 2:** Box–Behnken experimental design and observed responses.

Run	WPC (g)	Glycerol (g)	Sorbitol (g)	Grammage (g/m^2^)	Thickness (µm)	Peak Elongation (%)	Tensile Index (N.m/g)	Elastic Modulus (MPa)
1	3	1.9688	0.6563	129.10	94.15	28.62	0.55	9.53
2	3.5	1.9688	0.8063	142.27	113.80	21.06	0.80	13.97
3	3	1.9688	0.6563	129.10	94.15	28.62	0.55	9.53
4	2.5	1.7188	0.6563	104.97	83.19	21.02	0.90	19.01
5	2.5	1.9688	0.8063	115.69	90.56	31.50	0.48	6.99
6	3	2.2188	0.5063	124.77	98.21	19.64	0.75	15.22
7	3	1.7188	0.8063	113.70	94.29	30.51	1.20	28.22
8	3.5	2.2188	0.6563	138.78	121.38	30.31	0.63	11.78
9	3	1.9688	0.6563	129.10	94.15	28.62	0.55	9.53
10	3	1.7188	0.5063	98.28	97.20	18.11	1.35	29.36
11	3.5	1.7188	0.6563	127.12	101.39	32.42	1.45	34.45
12	3	2.2188	0.8063	131.41	104.05	31.58	0.50	8.30
13	2.5	1.9688	0.5063	103.50	76.63	32.67	0.63	10.99
14	3.5	1.9688	0.5063	133.37	114.00	21.71	0.93	21.25
15	3	1.9688	0.6563	129.10	94.15	28.62	0.55	9.53
16	3	1.9688	0.6563	129.10	94.15	28.62	0.55	9.53
17	2.5	2.2188	0.6563	126.43	83.71	33.59	0.48	8.55

**Table 3:** Chemical composition of fennel EO.

Retention Time (min)	Compounds	Relative Area (%)	Kovats’ Retention Index *	Chemical Family
12.70	Tricyclene	0.01	926	Monoterpene
13.51	α-Pinene	3.05	948	Monoterpene
13.72	α-Thujene	0.06	951	Monoterpene
15.65	α-Fenchene	0.03	953	Monoterpene
16.17	Camphene	0.31	956	Monoterpene
18.89	β-Pinene	2.31	964	Monoterpene
19.74	Sabinene	0.17	976	Monoterpene
22.63	β-Myrcene	2.02	991	Monoterpene
22.87	α-Phellandrene	4.48	1005	Monoterpene
23.90	α-Terpinene	0.03	1018	Monoterpene
25.39	Limonene	21.32	1031	Monoterpene
26.02	β-Phellandrene	0.59	1031	Monoterpene
25.25	1,8-Cineole	0.15	1031	Monoterpenic ether
27.79	*cis*-β-Ocimene	1.70	1041	Monoterpene
28.74	γ-Terpinene	0.39	1050	Monoterpene
29.03	*trans*-β-Ocimene	0.09	1062	Monoterpene
30.52	*p*-Cymene	1.25	1063	Monoterpene
31.52	α-Terpinolene	0.26	1063	Monoterpene
40.21	Fenchone	27.58	1096	Monoterpenic ketone
46.73	α-Copaene	0.06	1377	Sesquiterpene
48.54	Camphor	0.39	1518	Monoterpenic ketone
49.81	Linalool	0.01	1098	Monoterpenic alcohol
52.59	*trans*-α-Bergamotene	0.03	1438	Sesquiterpene
53.92	Terpinen-4-ol	0.11	1177	Monoterpenic alcohol
58.03	Estragole	5.90	1195	Phenylpropanoid
60.97	*D*-Germacrene	0.01	1482	Sesquiterpene
63.33	*cis*-Anethole	0.16	1209	Phenylpropanoid
66.45	*cis*-Sabinol	0.09	1139	Monoterpenic alcohol
67.56	*trans*-Anethole	24.81	1283	Phenylpropanoid
78.53	*p*-Anisaldehyde	0.25	1221	Aromatic aldehyde
84.12	Anis ketone	0.05	1384	Ketone
Total identified	31 compounds	97.67%	-	-

* Retrieved from National Institute of Standards and Technology, U.S. Department of Commerce, NIST Chemistry WebBook, SRD 69 (https://webbook.nist.gov/chemistry/name-ser/, accessed on 17 February 2023).

**Table 4:** Sequential model sum of squares and model summary statistics tested for responses.

Source	Sum of Squares	DF	Mean Square	*F*-Value	*p*-Value	R^2^	Adjusted R^2^	Predicted R^2^	PRESS	Remarks
Grammage										
Mean	2.61 × 10^5^	1	2.61 × 10^5^							
Linear	2014.03	3	671.34	19.57	<0.0001	0.8187	0.7769	0.6741	801.64	Aliased
2FI	45.99	3	15.33	0.38	0.7674	0.8374	0.7398	0.3875	1506.75	Aliased
Quadratic	324.29	3	108.10	10.00	0.0063	0.9662	0.9297	0.5077	1211.02	Suggested
Cubic	75.69	3	25.23							
Residual	0.00	4	0.00							
Total	2.63 × 10^5^	17	15,488.48							
Thickness										
Mean	1.60 × 10^5^	1	1.60 × 10^5^							
Linear	1852.95	3	617.65	31.51	<0.0001	0.8791	0.8512	0.7656	494.06	Aliased
2FI	163.83	3	54.61	6.00	0.0132	0.9568	0.9309	0.8559	303.83	Aliased
Quadratic	61.79	3	20.60	4.93	0.0379	0.9861	0.9683	0.7780	467.95	Suggested
Cubic	29.25	3	9.75							
Residual	0.00	4	0.00							
Total	1.62 × 10^5^	17	9534.81							
Peak elongation										
Mean	12840.85	1	12,840.85							
Linear	106.76	3	35.59	1.52	0.2257	0.2599	0.0891	−0.5341	630.28	Suggested
2FI	54.00	3	18.00	0.7197	0.5627	0.3913	0.0261	−2.0644	1258.96	Aliased
Quadratic	47.88	3	15.96	0.5596	0.6625	0.5078	−0.1249	−6.8745	3235.11	Aliased
Cubic	202.19	3	67.40							
Residual	0.00	4	0.00							
Total	13251.69	17	779.51							
Tensile Index										
Mean	9.71	1	9.71							
Linear	1.08	3	0.3607	10.10	0.001	0.6997	0.6304	0.5036	0.7676	Aliased
2FI	0.0426	3	0.0142	0.3367	0.799	0.7273	0.5636	0.1482	1.32	Aliased
Quadratic	0.4187	3	0.1396	320.33	<0.0001	0.9980	0.9955	0.9684	0.0488	Suggested
Cubic	0.003	3	0.001							
Residual	0.00	4	0.00							
Total	11.26	17	0.6623							
Elastic modulus										
Mean	3847.23	1	3847.23							
Linear	772.26	3	257.42	9.05	0.0017	0.6761	0.6014	0.4503	627.82	Aliased
2FI	48.31	3	16.10	0.5007	0.6902	0.7184	0.5495	0.0867	1043.16	Aliased
Quadratic	319.98	3	106.66	449.83	<0.0001	0.9985	0.9967	0.9767	26.56	Suggested
Cubic	1.66	3	0.5533							
Residual	0.00	4	0.00							
Total	4989.44	17	293.50							

**Table 5:** ANOVA and significance of regression coefficients.

Source	Grammage		Thickness		Peak Elongation		Tensile Index		Elastic Modulus	
	CE	*p*-Value	CE	*p*-Value	CE	*p*-Value	CE	*p*-Value	CE	*p*-Value
Model	129.100	0.0002	94.15	<0.0001	27.483	0.2557	0.550	<0.0001	9.530	<0.0001
X_1_	11.369	<0.0001	14.56	<0.0001	−1.660	0.3494	0.165	<0.0001	4.489	<0.0001
X_2_	9.665	<0.0001	3.91	0.001	1.633	0.3591	−0.318	<0.0001	−8.399	<0.0001
X_3_	5.394	0.0024	2.083	0.0236	2.815	1.1237	−0.085	<0.0001	−2.418	<0.0001
X_12_	−2.450	0.1798	4.868	0.0021			−0.100	<0.0001	−3.053	<0.0001
X_13_	−0.823	0.6332	−3.533	0.0106			0.005	0.6465	−0.820	0.0120
X_23_	−2.195	0.2236	2.189	0.0696			−0.025	0.0478	−1.445	0.0006
X_1_^2^	0.946	0.5734	1.789	0.1156			0.038	0.0078	0.971	0.0046
X_2_^2^	−5.721	0.0091	1.479	0.1813			0.278	<0.0001	7.946	<0.0001
X_3_^2^	−6.339	0.0055	2.809	0.0258			0.123	<0.0001	2.799	<0.0001

**Table 6:** Grammage, thickness, mechanical, and optical properties of the whey protein-based films.

Properties		Control Film	Film with Fennel EO	*p*-Values
Grammage (g/m^2^)		129.27 (113.49–136.40)	121.87 (111.56–134.99)	0.394
Thickness (µm)		94.00 (56.00–116.00)	84.50 (51.00–101.00)	0.058
Mechanical	Peak elongation (%)	14.03 (12.60–17.47)	31.61 (29.51–32.8)	0.014 *
	Tensile index (N.m/g)	0.40 (0.30–0.50)	0.50 (0.40–0.50)	0.034 *
	Elastic modulus (MPa)	14.86 (13.97–18.27)	10.38 (7.99–11.74)	0.155
Optical	Lightness (L)	33.22 (32.77–34.57)	32.16 (31.77–33.36)	0.199
	Redness (a)	−0.93 (−0.99;−0.87)	−0.95 (−1.01;−0.89)	0.630
	Yellowness (b)	1.35 (1.16–1.54)	1.53 (1.18–1.88)	0.520
	Transparency (%)	91.76 (91.52–92.48)	92.04 (91.76–92.88)	0.422

Results expressed as median and range; * Indicates a significant result (*p* < 0.05).

**Table 7:** Barrier properties of the whey protein-based films.

Barrier Properties		Control Film	Film with Fennel EO	*p*-Values
Water vapor	WVTR (g/m^2^.day)	183.66 (167.13–200.18)	200.50 (190.83–210.16)	0.490
	WVP (g/Pa.day.m) (×10^−5^)	1.22 (1.11–1.33)	1.35 (1.29–1.42)	0.432
Oxygen	OTR (cm^3^/m^2^.day)	5.13 (5.05–5.21)	35.94 (34.10–37.77)	0.038 *
	OP (cm^3^.µm/m^2^.day.kPa)	5.50 (5.00–6.00)	40.00 (35.00–45.00)	0.039 *
Oil	OilP (g.mm/m^2^.day)	7.55 (7.42–7.68)	6.89 (6.45–7.32)	0.357

Results expressed as median and range; * Indicates a significant result (*p* < 0.05).

**Table 8:** Contact angles and surface free energy of the whey protein-based films.

Properties	Control Film	Film with Fennel EO	*p*-Values
Water contact angle (°)	50.63 (47.15–51.99)	49.33 (48.30–50.07)	0.142
Diiodomethane contact angle (°)	74.03 (70.29–75.40)	68.58 (65.68–69.92)	0.009 *
Ethylene glycol contact angle (°)	82.67 (78.38–85.14)	81.39 (77.07–83.72)	0.377
Total surface free energy, ɤT (mN/m)	32.21 (31.35–35.07)	36.60 (35.18–38.02)	0.050
Polar component, ɤP (mN/m)	16.13 (14.65–17.61)	15.96 (14.82–17.10)	0.827
Dispersive component, ɤD (mN/m)	17.08 (15.99–18.17)	20.64 (19.80–21.48)	0.050

Results expressed as median and range; * Indicates a significant result (*p* < 0.05).

**Table 9:** Antioxidant and antibacterial activities of the whey protein-based films.

Properties		Control Film	Film with Fennel EO	*p*-Values
Antioxidant (% Inhibition)	β-carotene bleaching test	18.11 (17.76–24.24)	18.85 (17.56–23.74)	0.827
Antibacterial (Diameters of inhibition zones, mm)	*S. aureus* ATCC 25923	0	6 (Contact inhibition)	<0.001 *
	*L. monocytogenes* LMG 16779	6.35 (6.00–6.70)	6.85 (6.70–7.00)	0.368
	*E. faecalis* ATCC 29212	6 (Contact inhibition)	6.45 (6.00–6.90)	0.500
	*B. cereus* ATCC 11778	0	6 (Contact inhibition)	<0.001 *
	*S.* Typhimurium ATCC 13311	N.D.	N.D.	-
	*E. coli* ATCC 25922	6 (Contact inhibition)	6 (Contact inhibition)	1.000
	*P. aeruginosa* ATCC 27853	6 (Contact inhibition)	6 (Contact inhibition)	1.000

Results expressed as median and range; N.D.—not detected; * Indicates a significant result (*p* < 0.05).

## Data Availability

Data will be available by request to the corresponding authors.
